# Development of a dynamical statistical analog ensemble forecast model for landfalling typhoon disasters

**DOI:** 10.1038/s41598-023-43415-0

**Published:** 2023-09-27

**Authors:** Caiming Wu, Fumin Ren, Da-Lin Zhang, Jing Zhu, John Leonard McBride, Yuxu Chen

**Affiliations:** 1 School of Atmospheric Science, Nanjing University of Information Science and Technology, Nanjing, 210044 China; 2 State Key Laboratory of Severe Weather, Chinese Academy of Meteorological Sciences, Beijing, China; 3 Department of Atmospheric and Oceanic Science, University of Maryland, College Park, MD 20742 USA; 4 Xiamen Key Laboratory of Straits Meteorology, Xiamen Meteorological Bureau, Xiamen, 361012 China; 5 School of Earth Science, University of Melbourne, Melbourne, VIC Australia; 6 Research and Development Division, Bureau of Meteorology, Melbourne, VIC Australia; 7 Shantou Meteorological Bureau, Shantou, 515000 China

**Keywords:** Environmental social sciences, Natural hazards

## Abstract

In this report, the development of a Dynamical Statistical Analog Ensemble Forecast model for landfalling typhoon disasters (LTDs) and some applications over coastal China are described. This model consists of the following four elements: (i) obtaining the forecast track of a target landfalling typhoon, (ii) constructing its generalized initial value (GIV), (iii) identifying its analogs based on the GIV, and (iv) assembling typhoon disasters of the analogs. Typhoon track, intensity, and landfall date are introduced in GIV at this early development stage. The pre-assessment results show that the mean threat scores of two important damage levels of LTDs reach 0.48 and 0.55, respectively. Of significance is that most of the damage occurs near the typhoon centers around the time of landfall. These results indicate the promising performance of the model in capturing the main damage characteristics of typhoon disasters, which would help coastal community mitigate damage from destructive typhoons.

## Introduction

Typhoons, also referred to as tropical cyclones (TC) herein, are among the deadliest and costliest natural hazards with considerable economic loss every year in East Asia^1,2^. One example of costly TCs is Typhoon Rammasun (2014) that produced a direct economic loss of over ¥40 billion with more damage over coastal regions in Guangdong, Guangxi, and Hainan provinces^3^. Super-Typhoon Lekima (2019) was also one of the most serious typhoons to cause damage in China in recent years. In China, 11 provinces were affected, with a total of 14.02 million people affected and direct economic losses of ¥51.53 billion^4^. Because of the potential for more typhoon hazards under the current global warming trend^5–7^, and more rapid economic developments and population growth^8,9^, the typhoon-related disaster risk with more economic losses over coastal regions will likely continue to increase in the future^10–12^. Thus, it is highly desirable to improve our capability of disaster prevention and mitigation.

Skillful typhoon disaster pre-assessment, in which the property damage, potential casualties, and economic losses of a typhoon event that may occur can be evaluated, is of extreme importance for disaster prevention and mitigation^13^. After reviewing typhoon disaster pre-assessment models, Wu et al.^14^ categorized them into statistical, dynamical, and statistical–dynamical models. For the statistical models, many scholars paid attention to establishing mathematical relationships such as regression or exponent functions to pre-assess disaster risks and damage^15–19^. Some studies have integrated recent advances in fuzzy mathematics, grey theory and information spread technique in order to assess typhoon disaster risks^20,21^. With the rapid development of artificial intelligence, some neural network and support vector machine models have been developed^22–24^. This type of models, based on historical disaster information, makes direct connections between typhoon disaster and impact factors. Thus, these models are usually limited to regional applications, data accuracy, and oversimplified physical mechanisms of typhoon disaster.

The HAZUS Hurricane Model developed by Vickery et al.^25^ and the Florida Public Hurricane Loss Model (FPHLM) developed by Hamid et al.^26^, which can be considered as dynamical models, focus more on simulating and analyzing the dynamical processes of typhoon disasters with clear physical mechanisms. However, the complexity of typhoon disasters and the limitation of human’s knowledge on the disaster physical mechanism restrict the construction of typhoon disaster pre-assessment models at each phase, such as the description of the associated hazard scenarios, thereby making relatively large spreads of pre-assessment results.

Combining statistical methods to extract information from historical data to improve the forecasting skill of dynamical model forecasts has proven to be effective^27–29^. Currently, the idea is gradually used in the establishment of disaster pre-assessment model and appears to be promising for improving our capability of disaster prevention and mitigation^30,31^. These models make disaster pre-assessments directly by selecting historically similar typhoons that satisfy certain parameter threshold criteria for variables with physical characteristics. Nevertheless, we have not seen a well-recognized dynamical-statistical pre-assessment model in the literature.

Recently, Ren et al.^32,33^ developed the Dynamical-Statistical Analog Ensemble Forecast (DSAEF) theory that is based on relatively solid physical mechanisms for similarity forecast, and they also developed a technique to achieve the forecast, i.e., through the ensemble algorithms. A specific forecast model for a certain factor of prediction based on this theory can be built by taking into account the physical formation characteristics of the factor, combining with corresponding historical data, etc. The theory has been successfully applied to the precipitation forecast for landfalling typhoons, leading to the construction of the Dynamical Statistical Analog Ensemble Forecast Model for landfalling typhoon precipitation (DSAEF_LTP) with many encouraging results^34,35^. That is, three large sample-sized numerical experiments for precipitation forecasts of landfalling typhoons occurring in China^36–38^ using the most recent version of the model show better performance for ≥ 100 mm accumulated precipitation than the existing objective methods that are based on the current numerical weather prediction (NWP) models. Thus, the major objective of this study is to extend the encouraging results by applying the DSAEF theory to the pre-assessment for landfalling typhoon disasters (LTD) in an attempt to develop a DSAEF_LTD model. This will be done in conjunction with a complete typhoon disaster dataset at the county-level resolution from 1980 to 2018 over coastal China.

The next section introduces the DSAEF_LTD model. "Data and experimental design" describes the data and experimental design. "Results" analyzes the experiment results. A summary and discussion remarks are given in the final section.

## The DSAEF_LTD model

### Model description

The DASEF_LTD model is developed in accordance with the DSAEF theory. This model consists of the following four elements:

(i) Obtaining the track forecasts of a target landfalling typhoon by NWP models after the initial time T_0_, which is determined by parameter **P1** (see Table 1). The third column in Table 1 lists parameter values that can be selected for each experiment. For example, there are six options (values) for the initial time T_0_. The first option is 0000 UTC on the day of the typhoon-related heavy precipitation and maximum surface wind simultaneously occurring over land. A complete track of the target landfalling typhoon is the combination of the forecast locations after the initial time and the previously observed locations before and at that time.

**Table 1:** Parameters used in the DSAEF_LTD model and their value schemes.

Parameters (1–8)	Description	Number of schemes
Initial time (**P1**)	1: 12:00 UTC on Day1, 2: 00:00 UTC on Day1 3: 12:00 UTC on Day2, 4: 00:00 UTC on Day2 5: 12:00 UTC on Day3, 6: 00:00 UTC on Day3 (Day1: the first day of typhoon precipitation and wind occurs over land; Day2: the day before Day 1; Day3: the day before Day 2)	3 × 2 = 6
Similarity region (**P2**)	A parameter of the TSAI, which is a rectangular shaped region. Its southeastern vertex (C) can be the typhoon position at 0, 12, 24, 36 or 48 h before the initial time, and northwestern vertex (A) can be the typhoon position at 0, 6 or 12 h before the maximum lead time, the value of 1–15th are combined by C and A	5 × 3 = 15
Threshold of the segmentation ratio of a latitude extreme point (**P3**)	A parameter of the TSAI 1: 0.1, 2: 0.2, 3: 0.3	3
The overlapping percentage threshold of two typhoon tracks (**P4**)	A parameter of the TSAI 1: 0.9, 2: 0.8, 3: 0.7, 4: 0.6, 5: 0.5, 6: 0.4	6
Seasonal similarity (**P5**)	A parameter indicating the typhoon landfall date 1: whole year; 2: May–Nov; 3: Jul–Sept 4: the same landfall month as the target typhoon 5: within 15 days of the target typhoon landfall time	5
Intensity similarity (**P6**)	Four categories: 1: average intensity on the first impact day (i.e., the day typhoon precipitation and wind occur over land) 2: maximum intensity on the first impact day 3: average intensity on all impact days 4: maximum intensity on all impact days Five levels: 1: all grades, 2: the target typhoon intensity has the same grade or above the historical typhoon 3: the same grade or below, 4: only the same grade 5: the same grade or one grade difference	4 × 5 = 20
Number (**m**) of analog typhoons screened for the ensemble pre-assessment (**P7**)	1–10 for 1, 2, …, and 10, respectively	10
Ensemble pre-assessment scheme (**P8**)	1: mean 2: maximum	2
Total number of schemes	6 × 15 × 3 × 6 × 5 × 20 × 10 × 2 = 3,240,000
(ii) Constructing the generalized initial value (GIV) that includes not only the initial conditions at T_0,_ but also, if necessary, the subsequent temporal evolution of the main variables that may impact disaster occurrences in the dynamical and statistical sense. So, the GIV is distinguished from what is usually referred to as the initial value.
(iii) Identifying analogs based on the GIV. This step is to identify ***m*** top analogs according to the GIV. It consists of three substeps: identifying analogs for each variable, finding GIV’s analogs, and determining the ***m*** best analogs. The Track Similarity Area Index (TSAI) ^39^ is applied to the calculated area between the target typhoon track and a historical typhoon (i.e., since 1980) track in an established similarity region (**P2**). The threshold (i.e., **P3** in Table 1) of the segmentation ratio of a latitude extreme point represents the bend of typhoon tracks. The overlapping percentage threshold (i.e., **P4** in Table 1) of two typhoon tracks indicates the longitude and latitude overlap of two typhoon tracks. In other words, **P2**, **P3**, and **P4** determine the similarity of typhoon tracks between the target typhoon and its analogs. All historical typhoons are then sorted in an ascending order based on the TSAIs; the smaller the overlapped areas are, the higher is the similarity. The typhoon landfall season (i.e.. **P5** in Table 1) and typhoon intensity (i.e., **P6** in Table 1) similarities are further identified to eliminate the historical typhoons that differ significantly from the target typhoon in landfall time and intensity. Thus, determining several historical typhoons with the GIV that is the most similar to that of the target typhoon depends on the value of ***m*** according to **P7** (Table 1).
(iv) Assemble disasters of the analogs. The pre-assessment of potential disasters for the target typhoon is obtained by applying the best ensemble pre-assessment scheme P8 (Table 1) to the collected disasters from the remaining top ***m*** (**P7**) historical typhoons.

In addition, the third column in Table 1 gives the values of parameters that can be selected in the experiment. For example, there are six options (values) for the initial time (**P1**).

### Major model characteristics

Based on the above description, one can see the following three unique characteristics of the DSAEF_LTD model.

**Figure 1:**
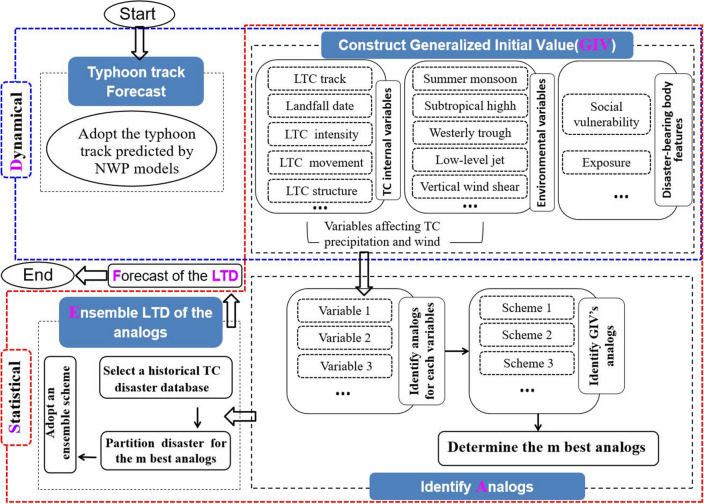
Flowchart of the DSAEF_LTD model in an ideal state, where typhoon track, typhoon landfall date, and typhoon intensity (see the top-right box) included in the GIV are currently selected.
  With the gradual introduction of typhoon hazard-related variables, the characteristics of the disaster-bearing bodies and artificial influences^40–44^ that contribute to the typhoon disasters can be progressively incorporated into the model.
(ii) The model uses the typhoon track forecasts from operational NWP models to determine the influences of target typhoons, given the revolutionary improvements in the operational track forecasts during the past few decades^45, 46^.
(iii) The typhoon hazard pre-assessment is achieved by using historically similar physical processes. Although the model itself does not include a specific physical expression of disaster processes, the associated disaster information of the most similar typhoons should be close to that of the target typhoons. This reflects the significance of historical observations in determining target typhoon disasters.

(i) Ideally, the GIV of DSAEF_LTD model involves typical four dimensions and may contain all possible variables associated with LTDs. It can be meteorological variables that related to heavy precipitation and high wind over land associated with typhoon disasters, e.g., typhoon movement, structure, and some environmental factors such as monsoonal flows, vertical wind shear, and subtropical high. It can also be some factors such as socioeconomic vulnerability, exposure to population and wealth, and disaster prevention and mitigation capability (see Fig. 1). In this study, typhoon track, landfall date, and intensity are chosen to construct the GIV at this development stage.

## Data and experimental design

### Data

To apply effectively the DSAEF_LTD model, it is essential to obtain a long historical record of meteorological observations (i.e., typhoon track, intensity, rainfall and winds), typhoon disaster information, and operationally available typhoon track forecasts.

**Figure 2:**
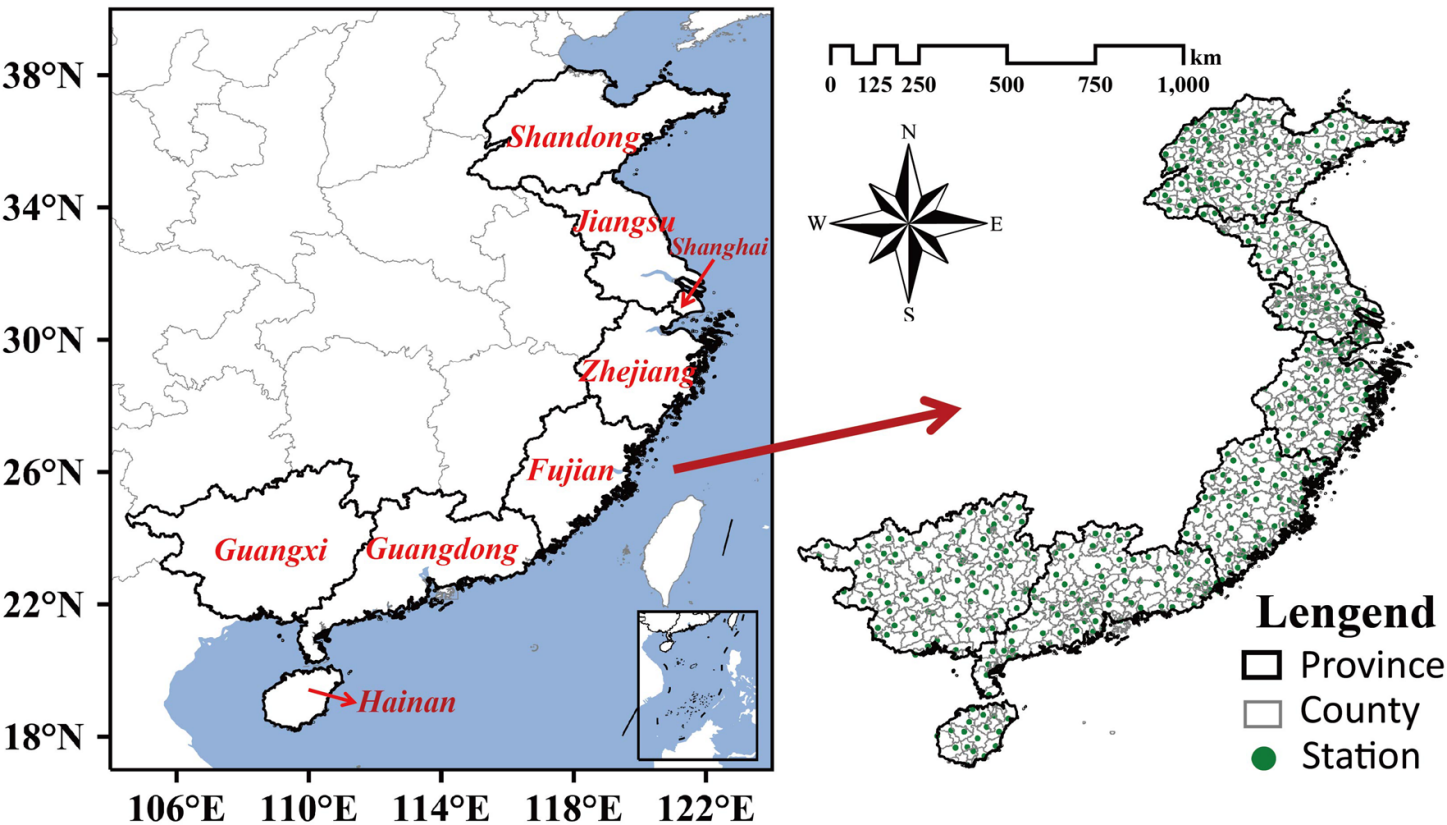
Spatial distributions of the 505 stations over the eight coastal provinces (i.e., Shandong, Jiangsu, Shanghai, Zhejiang, Fujian, Guangdong, Guangxi, and Hainan) of China (cited from Wu et al.^47^).

In this study, an integrated multi-source disaster dataset from 505 stations in China’s eight coastal provinces (see Fig. 2) is used, which consists of 442 typhoon disaster cases from 1980 to 2018. Each record of a typhoon disaster case includes the station name, disaster event and the corresponding damage in terms of direct economic losses. This long-period of dataset was developed from a reliable, available, and county-level disaster dataset from 2004 to 2013 by the National Climate Centre of China Meteorological Administration (CMA) . Specifically, typhoon precipitation and wind data over the shorter period are matched to disaster data and a functional relationship is derived between two factors and typhoon disaster (direct economic loss). This relationship is used to construct a disaster dataset for a longer period. What needs to be further explained is that the missing values of wind speed data reduced significantly after 1980, the difficulty for wind speed data acquisition and the complexity and time consumption of data processing restricted the time period of the study to 1980–2018^47^.

In addition, damage in terms of direct economic losses associated with each typhoon at individual stations was classified into the following five categories: mild, medium, heavy, severe, and extremely severe, which are denoted as C1–C5 in Table 2, respectively.

**Table 2:** Classifications for single station-based or typhoon case-based damage ( ¥0.1 billion, cited from Wu et al. ^47^).

Damage category	Mild (C1)	Medium (C2)	Heavy (C3)	Severe (C4)	Extremely severe (C5)
Single station-based	(0, 0.05]	(0.05, 0.10]	(0.10, 0.50]	(0.50, 2.55]	(2.55, –)
Typhoon case-based	(0, 7.42]	(7.42, 24.89]	(24.89, 85.29]	(85.29, 206.51]	(206.51, –)

The best track data are obtained from the Shanghai Typhoon Institute of CMA (https://tcdata.typhoon.org.cn/)^48,49^. This dataset includes the center location, maximum surface wind, and minimum sea-level pressure of each typhoon at 6-h intervals.

The forecast track and intensity data of each target typhoon is public released by CMA/National Meteorological Center and saved in the babj file. It is the result of a subjective revision based on various operational NWP models.

### Target typhoons

**Figure 3:**
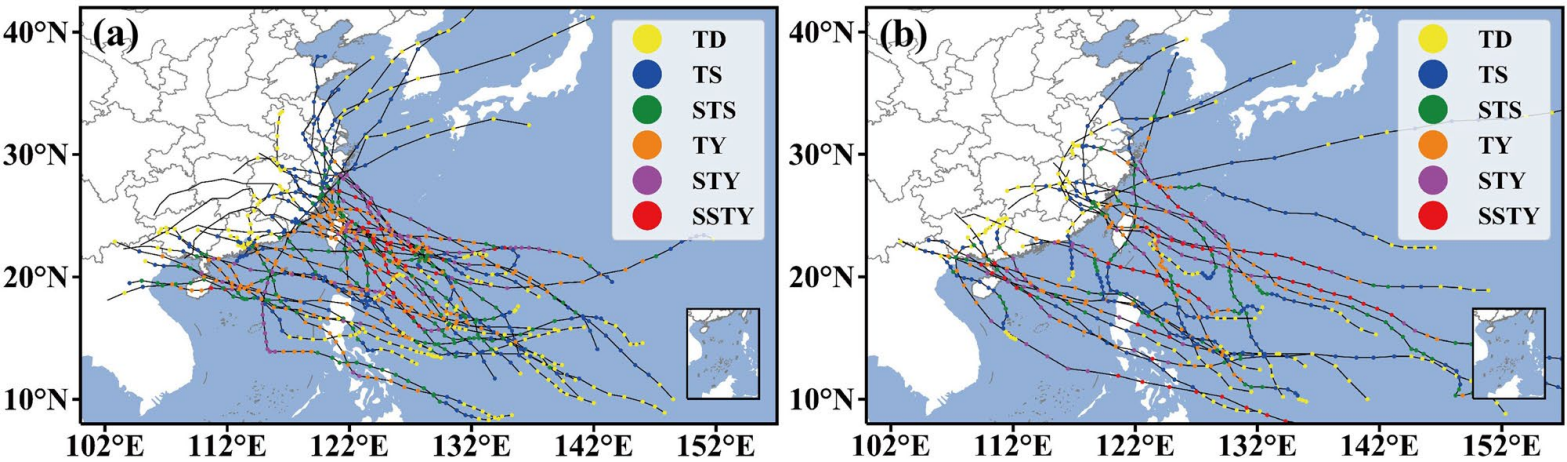
Tracks of target typhoons with varying intensities including (**a**) 30 training samples during 2004–2011; and (**b**) 21 independent samples during 2012–2015.

Target typhoons, consisting of training and independent samples for simulation and pre-assessment experiments, respectively, are determined by the following four criteria: (i) The typhoon damage reaches the C3 category and above because the pre-assessment accuracy for relatively heavy damage is of more concern to the public^50^. (ii) The same landfalling typhoon sample size from the eastern China and the southern China should be used for both the simulation and pre-assessment experiments in order to balance the influences of different large-scale flows on experiment results. (iii) All landfalling typhoon samples have forecast tracks. (iv) The training sample size should be larger than the independent sample size, and typhoons in the former should be relatively long-lived in order to ensure that the results are universally applicable. Meanwhile, considering the actual records for the period from 2004 to 2013^47^, as a result, 3 at C5, 16 at C4 and 11 at C3 damage category landfalling typhoons from 2004 to 2011 are selected as training samples (Fig. 3a), and 2 at C5, 12 at C4, 7 at C3 damage category landfalling typhoons from 2012 to 2015 as independent samples (Fig. 3b).

### Experiments for training and independent samples

As listed in Table 1, there are multiple values for each parameter. One value is taken from each of the eight parameters, together forming a pre-assessment scheme. So, there would be 3,240,000 different schemes to pre-assess the disasters of a target typhoon under idealized conditions. The key to the simulation experiments is to find the best pre-assessment scheme for the target typhoon disasters.

The procedures for determining the best pre-assessment scheme are given as follows: obtaining the common schemes from the simulation experiments that are suitable for all the 30 training samples, and then the best common scheme is identified from these schemes by some criteria. Due to the short track of some target typhoons that may limit the selection of similarity regions, or the small sample size of historically similar typhoons in the similarity region, some typhoons cannot take all the pre-assessment schemes. Thus, the common schemes of the 30 training samples are often less than 3,240,000.

### Effect evaluation methodology

The threat score (TS) is an important verification metric to evaluate the merits and demerits of the station-based damage pre-assessment ability of the DSAEF_LTD model. It is defined by

1 $$TS=\frac{hits}{hits+misses+false alarms}$$

where *TS* indicates the general pre-assessment ability ranging within [0, 1]; the closer to 1 is the higher pre-assessment ability; *hits* denotes the number of stations with correct forecasts whose observed and pre-assessed damage both reach a certain magnitude; *misses* refers to the number of stations whose observed damage reaches a certain magnitude but the pre-assessed damage does not; and *false* *alarms* is the number of stations whose pre-assessed damage reaches a certain magnitude but the observed damage does not.

The bias score is another indicator to test the station-based damage pre-assessment performance of the model, and to evaluate the tendency of false alarms or misses. It is defined by

2 $$BS=\frac{false alarms+hits }{misses+hits}$$

It is evident that *BS* = 1 when the area of false alarms is equal to the area of misses. The value is > 1 (< 1) if the area of false alarms is greater than (less than) the area of misses.

In this paper, TS scoring criteria are used to evaluate the simulation performance of each disaster distribution. In the simulation experiments, TSs of ≥ C3 and ≥ C2, which reflect the main damage characteristics of a typhoon disaster case and its overall disaster distribution, are selected to evaluate the simulation performance. TS_sum (i.e., TS of ≥ C3 plus TS of ≥ C2) is used to determine the best common scheme as the pre-assessment scheme.

Then, the pre-assessment experiments are conducted by applying the best common scheme to the 21 independent samples obtained during the years of 2012–2015. Like the calculation of TS_sum, BS_sum is obtained as BS of ≥ C3 plus BS of ≥ C2. TS_sum and BS_sum are both used to evaluate the performance of the DSAEF_LTD model in terms of pre-assessment ability.

## Results

### Simulation experiments

**Figure 4:**
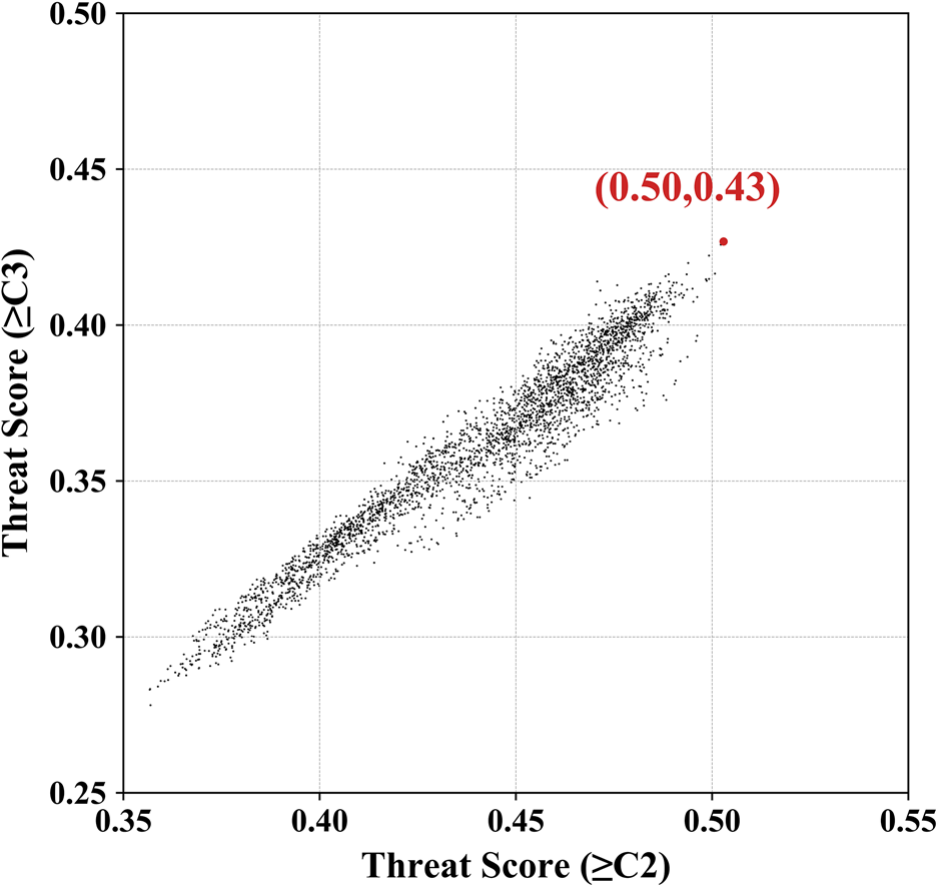
Threat scores of the 15,802 common schemes generated from simulation experiments, in which each point represents the effect of a simulated scheme. TS of ≥ C3 and TS of ≥ C2 represent the threat scores of the heavy damage and above, and the medium damage and above, respectively. The red dot denotes the best common scheme with the maximal TS_sum (i.e., TS of ≥ C3 plus TS of ≥ C2).

Based on the above discussion, 15,802 common schemes are found from the 30 training samples and then used for simulation experiments. Figure 4 shows the TS distribution of common schemes, with each point corresponding to a scheme. The red dot represents the best common scheme, at which TS_sum is maximized, i.e., with TS of ≥ C3 and TS of ≥ C2 being 0.43 and 0.50, respectively. The simulation performance for medium damage and above (i.e., ≥ C2) is slightly better than that for heavy damage and above (i.e., ≥ C3). This result indicates that the model has simulation ability in capturing the main damage characteristics of a typhoon disaster case and its overall disaster distribution, more effectively for ≥ C2 damage.

The parameter values of the best common scheme are as follows. The time of 0000 UTC on the first day that typhoon precipitation and wind occurred simultaneously over land is defined as the initial time of typhoon track forecast. A rectangle-shaped region, defined by typhoon locations at the initial time and 6 h before the maximum lead time, is used to determine the tracks of historically similar typhoons. The landfall times of the historically similar typhoons are limited to the months of July to September. In this study, the criterion for typhoon intensities could not be too strict, because the number of historically similar typhoons may be otherwise reduced, making the intensity parameter less effective for pre-assessment. Thus, selecting similar typhoons is less limited by typhoon intensity but more by typhoon track. For the damage of each station among these similar typhoons, the maximum damage value encountered is taken as a better ensemble pre-assessment scheme.

### Pre-assessment experiments

**Figure 5:**
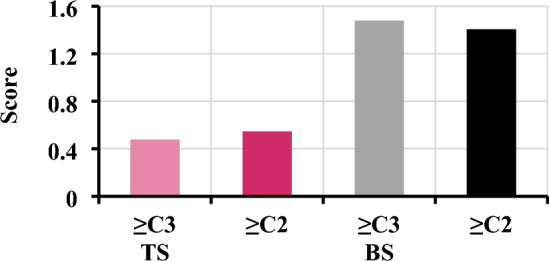
Average performance at the heavy damage and above level (i.e., ≥ C3), medium damage and above level (i.e., ≥ C2) in terms of threat scores (TS) and bias scores (BS) for the 18 independent samples taken during 2012–2015.

During the pre-assessment experiments, three independent samples are found to fail in applying the best scheme due to their short tracks; so, they are excluded from the analysis that follows. Figure 5 displays the mean scores of pre-assessment associated with the remaining 18 independent samples during different periods. Results show that TS of ≥ C3 and TS of ≥ C2 reach 0.48 and 0.55, respectively, during the years of 2012 to 2015. This indicates again that the model can capture the main damage characteristics of a typhoon disaster case. The BS during 2012–2015 shows that BS of ≥ C3 and BS of ≥ C2 reach 1.48 and 1.41, respectively, suggesting that the model is at a higher level of false alarms than misses. Based on the results, we may state that the DSAEF_LTD model has the general ability to pre-assess the two important damage levels, and that the pre-assessment errors resulted mainly from false alarms.

**Figure 6:**
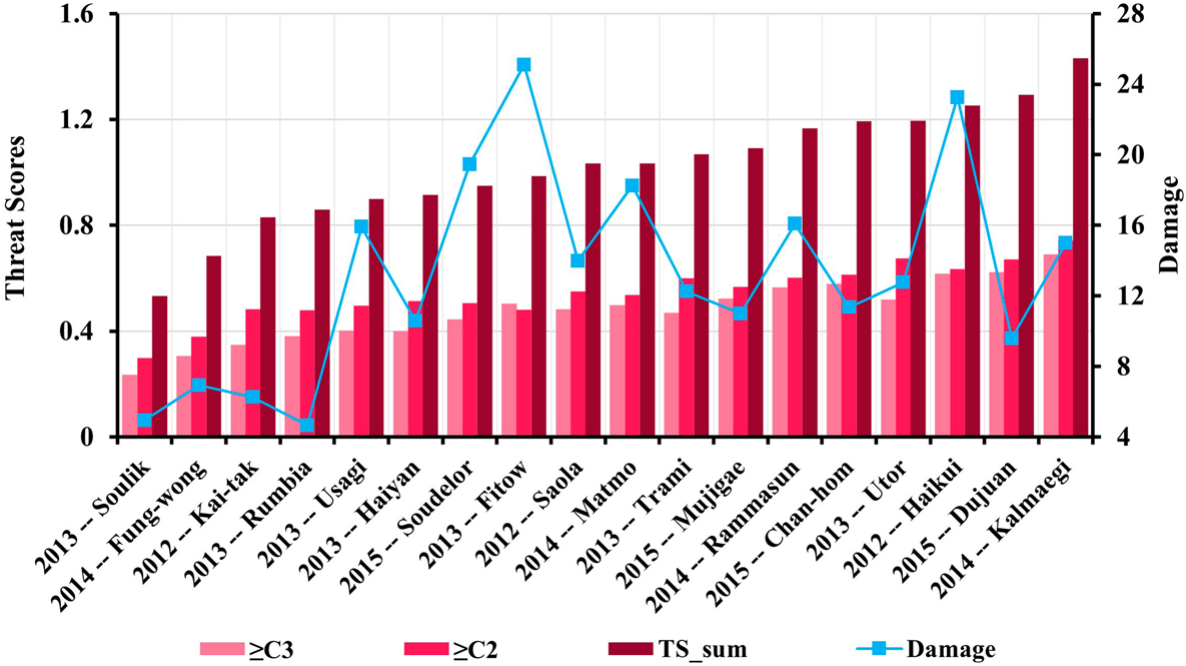
The DSAEF_LTD model pre-assessment of (**a**) TS of ≥ C3; (**b**) TS of ≥ C2; and (c) TS_sum (i.e., TS of ≥ C3 plus TS of ≥ C2) for each of the 18 typhoon disaster cases. Typhoon cases are listed in ascending order according to TS_sum scores. Blue points (curve) denote the observed damage (Unit: ¥1 billion) of individual typhoons.

Figure 6 shows TS of ≥ C3, and TS of ≥ C2 in relation to the observed damage associated with each of the 18 typhoon cases. In terms of TS of ≥ C3, the best pre-assessments are obtained for Kalmaegi (2014), Dujuan (2015), and Haikui (2012), with TS of 0.69, 0.62, and 0.61, respectively, and the worst pre-assessments are obtained for Kai-tak (2012), Fung-wong (2014) and Soulik (2013), with TS of 0.23, 0.31 and 0.35 in that order. The samples with the top three TS (≥ C2) are Kalmaegi (2014), Utor (2013), and Dujuan (2015), with TS of 0.74, 0.68, and 0.67, respectively. The three samples with the lowest TS (≥ C2) are Rumbia (2013), Fung-wong (2014), and Soulik (2013), with TS of 0.30, 0.38, and 0.48 in that order. For the typhoon disaster cases with the higher TS mentioned above, their economic loss amounts are all more than ¥8.5 billion, which are in the severe damage category and above. In contrast, the economic losses of typhoon cases with the lower TS are all less than ¥7 billion, corresponding to the heavy damage category. TS of ≥ C3 and TS_sum are well correlated with typhoon damage, with the correlation coefficients of 0.53 and 0.45, respectively. In fact, the result also depended on the selection criteria of the samples.The above results are encouraging, because they indicate that the DSAEF_LTD model could pre-assess well the main damage of more destructive typhoons.

### Analysis of representative cases

**Figure 7:**
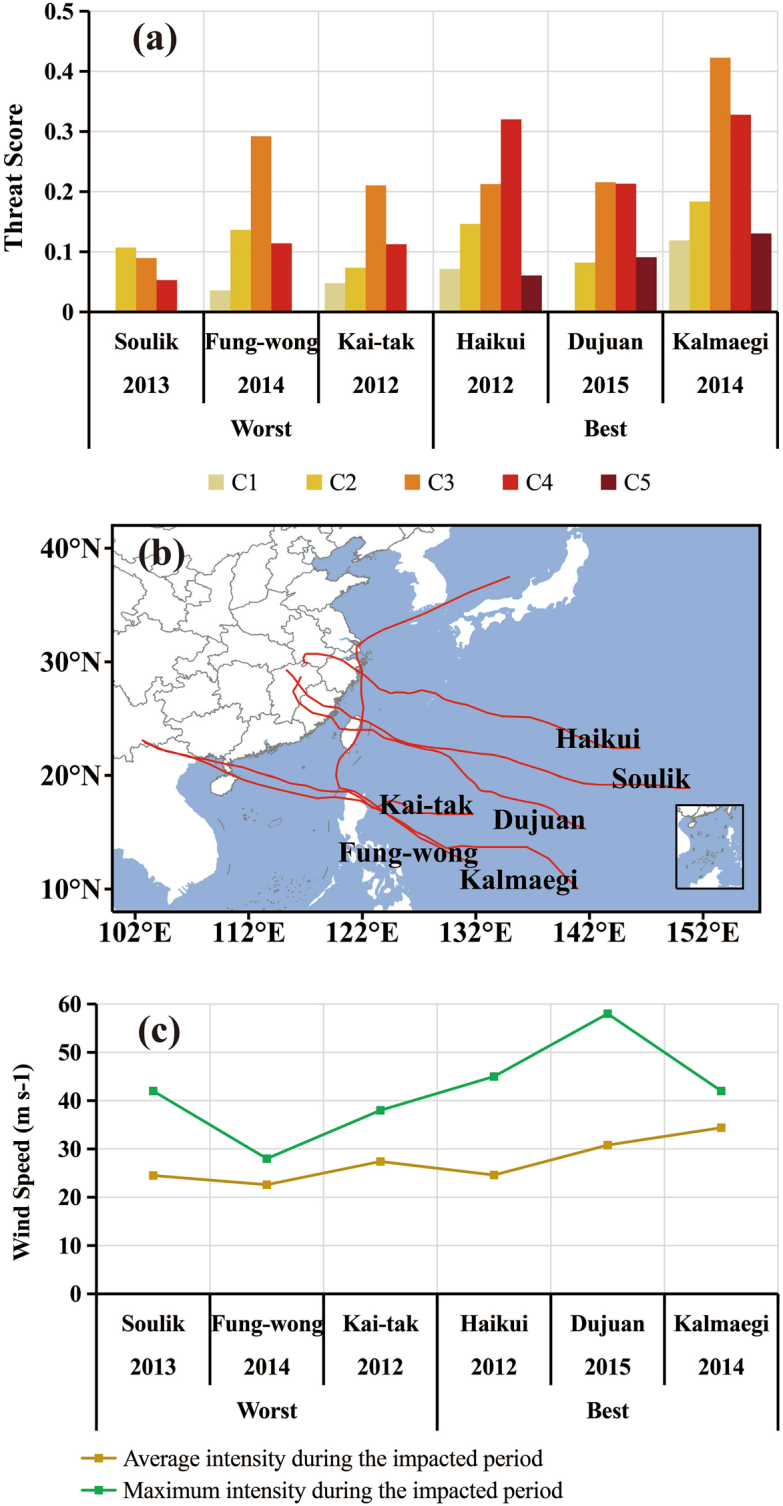
Comparison of two groups of typhoons with the respective best and worst pre-assessment performances. (**a**) TS at each damage category; (**b**) typhoon tracks; (**c**) typhoon intensity during the impacted periods.

To help gain insight into the pre-assessment capability of the DSAEF_LTD model, three typhoons with the highest TS_sum and three typhoons with the lowest TS_sum in the independent samples are compared in Fig. 7a. Generally, the model is capable of pre-assessing each damage category, especially for the heavy and severe damage categories. This is even true for Typhoon Fung-wong (2014) in the lowest TS_sum group. The model also exhibits some skills in pre-assessing the extremely severe damage, e.g., in association with Kalmaegi (2014), Dujuan (2015), and Haihui (2012). Unusual tracks of typhoons may affect the accuracy of pre-assessment results like Typhoon Kai-tak in 2012 (cf. Fig. 7a, b). In addition, large deviations from the most similar typhoon tracks and few optional historical similar typhoons, according to the TSAI index, both tend to reduce TS of the model pre-assessment.

Analysis of the different physical characteristics of the above two groups revealed that typhoon intensities had a greater impact on the pre-assessment results and the stronger the average intensity and maximum intensity of the typhoon during the impacted period, the better performance is the pre-assessment (Fig. 7c). This helps explain why some typhoons have similar tracks, but with different pre-assessments (cf. Fig. 7b, c). Based on the results from Fig. 6, we may see that the capability of the model in capturing disasters at the severe damage and above category by a relatively stronger typhoon is more pronounced. Note, though, that because the selected ensemble scheme takes the maximum value (i.e., **P8** in Table 3), the model tends to over-assess weak disaster cases to more severe damage categories.

**Table 3:** Optimized values of the best common scheme obtained from simulation experiments.

Parameter (1–8)	Optimized value
Initial time (**P1**)	2: 0000 UTC on the day of typhoon precipitation and wind occurring over land at the same time
Similarity region (**P2**)	6: typhoon locations at the initial time and 6 h before the maximum lead time as a rectangle
Threshold of the segmentation ratio of a latitude extreme point (**P3**)	3: 0.3
The overlapping percentage threshold of two typhoon tracks (**P4**)	2: 0.8
Seasonal similarity **(P5**)	3: Jul–Sept
Intensity similarity (**P6**)	(1 or 2 or 3 or 4, 1): Category: any Level: all grades
Number (**m**) of analog typhoons screened for the ensemble pre-assessment (**P7**)	2
Ensemble pre-assessment scheme (**P8**)	2: the maximum damage value

**Figure 8:**
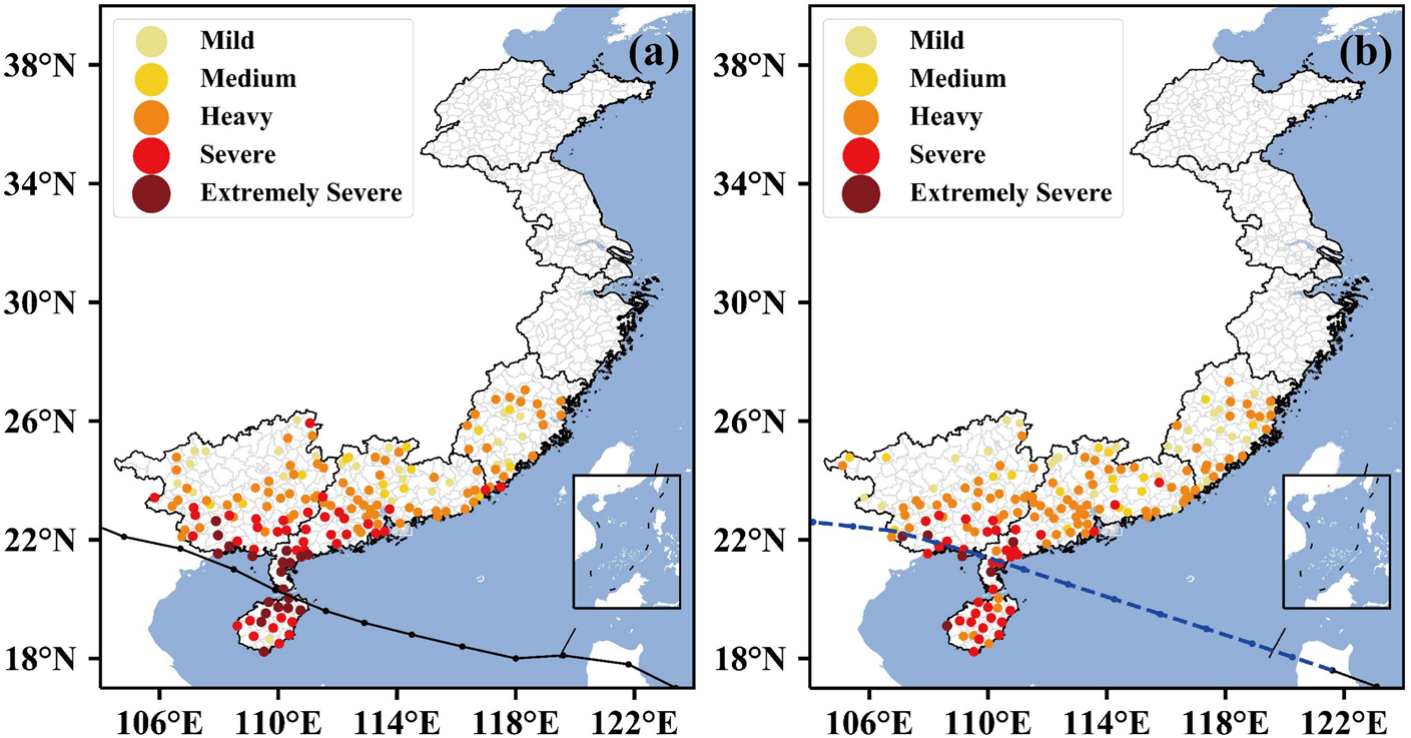
Spatial distribution of the damage (¥0.1 billion) associated with Typhoon Kalmaegi (2014). (**a**) Observations; and (**b**) Pre-assessments. The observed (in black) and forecast (in blue) tracks are given in (**a,b**), respectively.

Typhoon Kalmaegi (2014), which has the highest TS_sum value in the pre-assessment experiments, is used to demonstrate how well the observed spatial damage distribution could be pre-assessed. The observed damage distribution, given in Fig. 8a, shows decreasing damage extents laterally outward from the typhoon tracks around the landfall time. So, the typhoon damage decreases from the coastal to inland regions over southern China, i.e., mainly occurring in northern Hainan, southern Guangxi, and eastern Leizhou Peninsula (southwestern Guangdong). A comparison of Fig. 8a, b reveals that the DSAEF_LTD model reproduces quite well the spatial distribution of each damage category, especially for the main damage areas. This result is as expected because of the relatively accurate track forecast of the typhoon. However, some deviations from the observed damage appear in the eastern and northeastern coastal areas of the Leizhou Peninsula, the southern part of Guangxi, and the western part of Hainan, where the model under-assesses the extremely severe damage at many stations (cf. Fig. 8a, b). This may be attributed to the forecast errors of typhoon intensity whose research progress has remained stagnant^51, 52^. Despite the presence of these errors, this case analysis indicates that the DSAEF_LTD model could be an important tool for pre-assessing the overall disaster distribution of a typhoon disaster case, especially for the heavy damage occurring in the core region of a typhoon prior to and after landfall.

## Summary and discussion

In this paper, we present the development of a Dynamical Statistical Analog Ensemble Forecast model for landfalling typhoon disasters (DSAEF_LTD) in order to improve our capability of disaster prevention and mitigation. Disaster-damage simulation experiments using this model are then conducted with 30 landfalling typhoons occurring during 2004–2011 as training samples, followed by disaster-damage pre-assessment experiments with 18 landfalling typhoons occurring during 2012–2015 as independent samples. Major findings are summarized as follows:

(i) The DSAEF theory can be successfully applied to pre-assessing landfalling typhoon disasters in coastal China. The DSAEF_LTD model consists of the following four elements: obtaining the forecast track of a target landfalling typhoon; constructing the generalized initial value (GIV); identifying analogs based on the GIV, and assemble typhoon disasters of the analogs. In this early stage of model development, typhoon track, landfall date, and intensity are considered into GIV. In addition, in the fourth elements of the model composition, an integrated multi-source disaster dataset from 505 stations in China’s eight coastal provinces is used for the model establishment.
(ii) The DSAEF_LTD model is tested with 15,802 common schemes through the simulation experiments. The best common scheme is then identified in accordance with the highest threat score from all the training samples. The best scheme, containing 8 parameters, uses the initial time at 0000 UTC on the first day that typhoon precipitation and maximum surface wind simultaneously occur on land, and the maximum damage value as the damage pre-assessment scheme.
(iii) An application of the best common scheme through the pre-assessment experiments shows that threat scores of the heavy damage and above, and the medium damage and above for the independent samples reach 0.48 and 0.55, respectively. This indicates the ability of the DSAEF_LTD model in capturing the main damage characteristics of typhoon disasters, especially for more destructive ones. In addition, the model could reproduce the general distribution of damage, namely, from the core region over coastal regions laterally outward to inland around the landfall of a typhoon.

Despite the encouraging application of the DSAEF_LTD model, some pre-assessment errors are noted, suggesting that more improvements of the model are much needed. New variables in meteorology, such as landfalling typhoon structures, monsoonal surge, cloud physical properties and other factors should be considered in GIV. Many research indicated that the disaster-related typhoon wind and rainfall characteristics are related to these factors^53–56^. In addition, considering the complexity of disaster formation, the factors related to environmental vulnerability, economical, agricultural and industrial developments should also be considered. How to express these physical factors objectively is worth exploring. The impact of each factor incorporation on model pre-assessment performance also needs to carry out a large number of sample experiments in the future. Meanwhile, more tests need be conducted in the future to determine whether the existing parameters selected herein are suitable for other regions during the landfall of TCs. Clearly, all these improvements will help communities make an informed decision for mitigating damage and casualties from destructive TCs.

Nowadays, the pre-assessment results of previous models concentrated more on damage of one particular province or the total damage of a typhoon, no models could give the county-level resolution damage of enough provinces for comparison^14^, so the pre-assessment results were only compared with observations in this study. Meanwhile, the model has shown good performances for the typhoons at C5 damage category, such as Typhoon Haikui (2012) and Typhoon Fitow (2013). However, for other typhoons at C5 category, the simulation or pre-assessment capabilities of the model need to be further tested in the future with the update and expanstion of the typhoon disaster dataset.

## Data Availability

The typhoon data used in this study are the best-track tropical cyclone datasets from Shanghai Typhoon Institute, which can be obtained from http://tcdata.typhoon.org.cn/. Daily precipitation data and hourly wind data during the period 1980–2018 over the 505 stations of coastal area in China are obtained from the National Meteorological Information Center. Due to the data privacy policy of research institutions, these data are not publicly available. Original Disaster data for China’s coastal area from 2004 to 2013 are obtained from the National Climate Center; because of the data privacy policy of research institutions, these data are also not public. The integrated dataset can be acquired from the corresponding author.
