# Author Correction: Development of a dynamical statistical analog ensemble forecast model for landfalling typhoon disasters

**DOI:** 10.1038/s41598-024-61676-1

**Published:** 2024-05-17

**Authors:** Caiming Wu, Fumin Ren, Da-Lin Zhang, Jing Zhu, John Leonard McBride, Yuxu Chen

**Affiliations:** 1 School of Atmospheric Science, Nanjing University of Information Science and Technology, Nanjing, 210044 China; 2 State Key Laboratory of Severe Weather, Chinese Academy of Meteorological Sciences, Beijing, China; 3 Department of Atmospheric and Oceanic Science, University of Maryland, College Park, MD 20742 USA; 4 Xiamen Key Laboratory of Straits Meteorology, Xiamen Meteorological Bureau, Xiamen, 361012 China; 5 School of Earth Science, University of Melbourne, Melbourne, VIC Australia; 6 Research and Development Division, Bureau of Meteorology, Melbourne, VIC Australia; 7 Shantou Meteorological Bureau, Shantou, 515000 China

Correction to: *Scientific Reports* 10.1038/s41598-023-43415-0, published online 27 September 2023

The original version of this Article omitted an affiliation for Caiming Wu. The correct affiliations are listed below.

School of Atmospheric Science, Nanjing University of Information Science and Technology, Nanjing, 210044, China.

State Key Laboratory of Severe Weather, Chinese Academy of Meteorological Sciences, Beijing, China.

Also author Fumin Ren was incorrectly affiliated with ‘School of Atmospheric Science, Nanjing University of Information Science and Technology, Nanjing, 210044, China’. The correct affiliation is listed below.

State Key Laboratory of Severe Weather, Chinese Academy of Meteorological Sciences, Beijing, China.

The original Article has been corrected.

